# Collaboration in teams with nurse practitioners and general practitioners during out-of-hours and implications for patient care; a qualitative study

**DOI:** 10.1186/s12913-017-2548-x

**Published:** 2017-08-23

**Authors:** Mieke van der Biezen, Michel Wensing, Lusine Poghosyan, Regi van der Burgt, Miranda Laurant

**Affiliations:** 1 Radboud University Medical Center, Radboud Institute for Health Sciences, Scientific Center for Quality of Healthcare, IQ Healthcare, P.O. Box 9101, 6500 HB, Nijmegen, The Netherlands; 2 Department of General Practice and Health Services Research, Health Services Research and Implementation Science, Heidelberg University, Marsilius Arkaden-Turm West, Im Neuenheimer Feld 130.3, 69120 Heidelberg, Germany; 3 Columbia University School of Nursing, 168th St., Suite 219, New York, NY 10032 USA; 4 Foundation for Development of Quality Care in General Practice, Tilburgseweg-West 100, 5652 NP, Eindhoven, The Netherlands; 5 HAN University of Applied Sciences, Faculty of Health and Social Studies, P.O. BOX 6960, Nijmegen, 6503 GL The Netherlands

**Keywords:** Collaboration, Inter-professional relations, Nursing, Nurse practitioner, Out-of-hours care, Primary care, Skill mix

## Abstract

**Background:**

Increasingly, nurse practitioners (NPs) are deployed in teams along with general practitioners (GPs) to help meet the demand for out-of-hours care. The purpose of this study was to explore factors influencing collaboration between GPs and NPs in teams working out-of-hours.

**Methods:**

A descriptive qualitative study was done using a total of 27 semi-structured interviews and two focus group discussions. Data was collected between June, 2014 and October, 2015 at an out-of-hours primary care organisation in the Netherlands. Overall, 38 health professionals (GPs, NPs, and support staff) participated in the study. The interviews were audio-taped and transcribed verbatim. Two researchers conducted an inductive content analysis, involving the identification of relevant items in a first phase and clustering into themes in a second phase.

**Results:**

The following four themes emerged from the data: clarity of NP role and regulation, shared caseload and use of skills, communication concerning professional roles, trust and support in NP practice. Main factors influencing collaboration between GPs and NPs included a lack of knowledge regarding the NPs’ scope of practice and regulations governing NP role; differences in teams in sharing caseload and using each other’s skills effectively; varying support of GPs for the NP role; and limited communication between GPs and NPs regarding professional roles during the shift. Lack of collaboration was perceived to result in an increased risk of delay for patients who needed treatment from a GP, especially in teams with more NPs. Collaboration was not perceived to improve over time as teams varied across shifts.

**Conclusion:**

In out-of-hours primary care teams constantly change and team members are often unfamiliar with each other or other’s competences. In this environment, knowledge and communication about team members’ roles is continuously at stake. Especially in teams with more NPs, team members need to use each other’s skills to deliver care to all patients on time.

## Background

The increasing demand for out-of-hours primary health care and the shortage of general practitioners (GPs) in this setting requires the development of innovative models of care delivery to meet the needs of all patients [1–3]. As in most western countries, out-of-hours care in the Netherlands is delivered by large scale general practitioner cooperatives (GPC) in which GPs from a region have duties to take care of the population when regular hours care is not available [3]. Internationally, as well as in the Netherlands, a growing number of out-of-hours care delivery models provide care to patients in teams with both GPs and nurse practitioners (NPs) [4, 5]. Globally, advancing nursing roles in health-care delivery and promoting task-shifting has been supported by policy makers, and regulatory and educational reforms [6]. Task-shifting between NPs and GPs is being increasingly implemented to help meet the demand for primary care and reduce the work burden of GPs. Such care models enable GPs to effectively utilise their training and experience by focusing on caring for the most vulnerable and complex patients [7, 8].

Nurse practitioners are capable of providing 67–93% of all primary care to patients, including those who seek care during out-of-hours, given their training and clinical expertise [9]. Although extant studies show positive outcomes for care delivered by NPs [10, 11], there are some concerns regarding the deployment of NPs in primary care. Currently, most studies of the involvement of NPs in primary care have focused on care delivered during regular office hours, with limited attention to NP care out-of-hours. To date, we do not have evidence about NP deployment in out-of-hours primary care and its impact for team collaboration.

Team-based care delivery models are key aspects of health care reforms aimed at improving access to care and patient outcomes. Collaboration in such teams involves care providers caring for groups of patients independently and interdependently, supporting each other to fully use their separate and shared skills [12]. Facilitating collaboration between NPs and GPs can have significant consequences for patient care. Schadewaldt, McInnes, Hiller and Gardner [13] identified 20 barriers and facilitators to collaboration between NPs and GPs in primary care in a systematic review of 27 papers. Examples of barriers and facilitators include clarity of the NP role, complementary practice ideology, time to collaborate and financial support. Although these factors give a broad understanding of collaboration between NPs and GPs, it is questionable whether all factors also apply to collaboration in out-of-hours care as in such settings GPs and NPs rotate in every shift. During regular office hours, in 81% of the Dutch general practices, GPs work alone or with one other GP [14]. In contrast, there are 50 to 250 GPs affiliated with each GPC sharing shifts to deliver out-of-hours care. Fulltime GPs are, on average, on call twice a month to work six to eight hour shifts each time to deliver care during the evening, night or weekend [15, 16]. Therefore, team members at the GPC differ in every shift and are often unfamiliar with each other and with each other’s competencies. Furthermore, they are unlikely to know the patients seeking out-of-hours care and do not have access to patients’ medical records prior and during the encounter. Research shows that collaborative practice takes time, and unfamiliarity with each other’s skills and competencies constrains team collaboration [13, 17, 18]. In addition, in stable teams, NPs and GPs develop favourable collegial relationships over time. Thus, lack of opportunities to work together and unfamiliarity between the team members may negatively affect collaboration, further limiting the effective deployment of NPs in out-of-hours care. It is known that ineffective communication and collaboration in teams negatively influences patient outcomes [19, 20]. This study will contribute to the evidence base for guiding optimal team structures with NPs and GPs in out-of-hours primary care.

## Methods

### Aim

The aim of the study was to identify the factors influencing collaboration between GPs and NPs in out-of-hours teams.

### Design

This study was part of a large research project that had two components. The first component included a quasi-experimental study that compared different teams providing out-of-hours care (ClinicalTrials.gov ID NCT02407847) [21]. The second component, which included a qualitative study is presented in this paper. This descriptive qualitative study was focused on team members’ views regarding factors influencing collaborative practice between GPs and NPs in out of hours. The consolidated criteria for reporting qualitative research (COREQ-32) were used to design and report the study.

### Setting and participants

The study was conducted at a GPC in the South East Netherlands. The GPC and the hospital emergency department (ED) share an emergency care access point for self-referred patients, but they operate independently [22]. The GPC, with its 160 GPs from the region, serves an area with a population of approximately 304,000 inhabitants. The GPC has been operating since 2001, and more than half the GPs have been affiliated with the GPC from the start or within the first year of the GPCs’ operation. At the start of the study, the mean age of the participants was 47.5 years (SD 9.7) and 50.3% of them were male.

Since 2011, this GPC has employed NPs to deliver out-of-hours care. The 10 NPs employed during the study period had an average of 1.8 years (SD 1.2) experience as an NP and had worked at the GPC for an average of 1.6 years (SD 1.1). At the start of the study, NPs’ mean age was 45.2 years (SD 9.4) and only one male NP. In the Netherlands, NPs have the authority to independently initiate and perform reserved medical procedures (e.g. puncture, prescribing medicines and simple surgical procedures) in his/her area of expertise using the same guidelines as GPs [23, 24]. In addition to this national authority, before implementing the NP role, the GPC formulated a scope of practice for the NPs based on NPs’ education and training. The following patients were defined by the GPC as being outside an NPs’ scope of practice: patients younger than one year or patients suffering psychiatric complaints, abdominal pain, chest pain, a neck ailment, headache, or dizziness. All other patients were within an NP’s scope of practice. The GPC distributed the list of patients who were excluded from NP care on the intranet. In addition to the online communication NPs were asked to inform GPs about the excluded patient groups at the start of every shift. Care providers were not offered other training regarding collaboration and team effectiveness during or after the implementation of the NP.

All patients who seek acute care out-of-hours can contact the GPC call centre by a single, regional telephone number. At the call centre triage nurses allocate patients for several GPC locations to an appropriate care pathway based on the risk stratification. All patients who will attend for a consultation at the GPC are scheduled on a presentation list. The triage nurses at the call centre are not part of the team that provides care at the GPC. Moreover, they are not aware of the team structure regarding the ratio of GPs and NPs during our study. After the triage, the patients scheduled for consultation can visit the GPC location in their region. Based on the scheduled time, patients’ urgency levels and complaints GPs and NPs call the waiting patients in for consultation [25]. The team as a whole is responsible for providing care to all scheduled patients on the presentation list.

Data were collected from two teams with different structures. Team-A comprised three GPs and one NP. Team-B comprised two GPs and two NPs. Both teams were supported by one receptionist and one medical assistant. In the Netherlands, medical assistants have followed four years of education and in primary care they perform routine diagnostic and therapeutic interventions and are the patients’ first point of contact for health education and the booking of practice visits [26].

The same individual team members (GPs, NPs, medical assistants, and receptionists) could work in Team-A in one shift and in Team-B on the next shift. Eligible participants included all team members who worked a shift either in team-A or team-B. Participants were invited by the primary researcher or the manager of the GPC by e-mail or telephone to participate, and they were informed of the details of the study.

### Data collection

Data were collected between June, 2014 and October, 2015. First, we conducted semi-structured individual interviews. The interview days were planned in advance, and the researchers were unaware which individuals would be part of the team that day. Prior to the interview, participants were asked for written and/or oral informed consent.

Face-to-face interviews were conducted with GPs, NPs, medical assistants and receptionists directly after the shift at the GPC, or by telephone at the first availability of the participants. Teams were interviewed at least during four weekend days with different team members. Data analyses took place concurrently with the interviews to explore emergent themes and determine data saturation. After the first interviews, if necessary more interviews were conducted until researchers agreed data saturation was reached.

Second, in addition to the interviews, focus group discussions were conducted to explore questions arising from the interviews to further enrich the data. Focus group discussions were held with NPs only. GPs conduct only a few shifts per year at the GPC, wherefore they do not have much experience in working with NPs at the GPC. Data saturation about their experiences with NP collaboration was reached during the individual interviews. NPs work at least one shift a week at the GPC, and individual interviews did not produce sufficient information to get in-depth insight into collaboration during the shifts. During the focus group interviews, NPs were encouraged to talk with each other and share their experiences and perspectives on team collaboration. The focus group discussions were held in February, 2015 and May, 2015 at the office of the Foundation for Development of Quality Care in General Practice.

The individual interviews and focus group discussions were conducted by MB and RB, both female health scientists. The participants did not know the researchers prior to the study. Field notes were taken during the data collection. The interviews and focus groups were audio-taped and then transcribed verbatim. Data were anonymized and kept confidential.

#### Interview guide

The interview guide was developed by the primary researcher (MB) with guidance from the co-authors (ML, RB). Three main topic areas were included in the guide: experiences of working in collaborative practice, barriers and facilitators regarding collaborative practice, and the implications of collaborative practice for patient care. Each topic area included two or three open-ended questions to encourage participants to discuss their perspectives and considerations about their experience, workload, professional routines, communication, work agreements, and patient care. The discussion guide for the focus groups included the same topics. Because the NPs who participated in the individual interviews also participated in the focus groups, results from the individual interviews were used as a starting point for the focus group discussion. Participants were encouraged to reflect on the findings and discuss NP and GP collaboration during shifts and how it impacts patient care.

### Data analysis

First, transcribed interviews and focus group data were coded inductively. Coding was done with constant discussion of interpretations by two researchers (MB, IM), guided by the research aim, resulting in one joint codebook. The codes for both teams were then grouped into overall themes at a higher level of theoretical abstraction. Data were declared to have reached saturation when, in both teams, no new codes were emerging. Atlas.ti software V.7.1.5. was used to analyse the data. Interpretation of the results and assessment of data saturation started during data-collection by the two researchers and the project leader (MB, ML, RB). An independent workgroup of NPs, GPs and medical assistants provided feedback on the preliminary findings, which was used to sharpen the analysis. The final elicitation of themes and categorization of interview material into themes was performed by three researchers (MB, ML, LP).

## Results

All invited participants agreed to participate in the interviews. A total of 27 interviews were conducted, 12 in team-A and 15 in team-B. In addition, 11 NPs participated in the two focus group discussion. Due to the small number of NPs at the GPC, four NPs participated both in the individual interviews and in the focus group discussions (see Table 1). The interviews took, on average, 21 min; the focus groups, on average, 60 min.

**Table 1:** Participating team members

	Team-A	Team-B	Focus group	Total
N Receptionists	2	2		4
N Medical assistants	2	3		5
N Nurse Practitioners	2	2	11	15^a^
N General Practitioners	6	8		14
*Total*	*12*	*15*	*11*	38

^a^Due to the small number of NPs, four NPs are included in both the individual interviews as in the focus groups

Four themes influencing collaboration in out-of-hours care delivery emerged from the analysis. (see Table 2).

**Table 2:** Empirically-derived themes influencing collaboration in out-of-hours primary care

Theme 1. Clarity of NP role and regulation	
Theme 2. Shared caseload and use of skills	
Theme 3. Communication concerning professional roles	
Theme 4. Trust and support in NP practice	

### Theme 1. Clarity of NP role and regulation of NP practice

Regardless of the team structure, there was a general lack of awareness among GPs about NPs’ scope of practice. The GPC formulated the role of the NP in writing, and provided a list on the intranet of those patients who are excluded from NP care. However, in contrast to the NPs, none of the GPs knew about the document or its exact content. Most GPs believed that NPs are not allowed to see complex patients, but they lacked specific knowledge or had misconceptions about the specifics regarding which patients NPs were unable to see. This is illustrated by a GP from team-A: “I know they don’t treat complex patients, or no abdominal pain… I think I know it.” An NP in the focus group stressed the importance of communicating clearly with GPs regarding the NP scope of practice: “It’s important to be very specific to GPs about what patients we don’t treat. Often they say ‘Oh yeah, I know,’ but turns out they don’t know it at all.”

NPs and medical assistants reported that NPs sometimes treated patients from the excluded patient groups, such as patients younger than one year, when they had the knowledge and expertise to work with these patients. A medical assistant from team-B explained: “The exact scope of the NP is sometimes handled a bit flexibly. NPs might call in patients with, for example, abdominal pain in case it looks like a bladder infection.” Some GPs found it confusing that different NPs treat different patients.

Although legal regulations regarding NP care were not clear for many GPs (e.g. some had the belief they needed to authorise NPs’ drug prescriptions), none of the GPs expressed concerns about legal liability for the NP care. A GP from team-B said: “My knowledge about the role of the NPs is somewhat diluted. Also, some NPs treat different patients than others. I believe I have to authorise NPs’ drug prescriptions as well.” Lastly, most GPs said they did not have prior experience working with NPs in their practice and were uncertain whether they had ever worked with NPs at the GPC before.

### Theme 2. Shared caseload and use of skills

In team-A, none of the GPs reported differences in the number of patients and type of patients they treated between teams working in teams with and without NPs. Moreover, although some patients were excluded for NP care, none of the GPs changed his or her professional routine based on the NPs’ scope of practice or in the type of patients he or she cared for. One GP from team-A said: “I started my day with caring for the first patient on the presentation list. I didn’t pay attention to the NP really.”

In team-B, GPs generally stated they had to treat more complex patients compared to working in teams with only GPs. However, there were differences between GPs. One GP from team-B said: “I think the consultations become more intense and difficult; the complaints are more complex.” Another GP from team-B commented; “I think there is not really a difference in the type of patients I treated today, I saw everything.”

In Team-B most GPs said they changed their professional routines by providing care for more complex patients (especially those with abdominal complaints), in order for the NPs to focus on less complex patients. A GP from team-B explained:

If there are two NPs we have to check all patients on the presentation list, not only their urgency level, but also their complaint. If it’s, for example, an ear infection, I take the next patient and leave the ear infection to the NP. Checking who the patients are on the presentation list is less important in shifts with three GPs.

Both NPs, medical assistants and receptionists indicated differences between GPs regarding changing their routines in teams with NPs. An NP from team-B commented: “At times I see GPs treating patients that could have been treated by us, while other patients from the presentation list are waiting. Not every GP is willing to cooperate.” General practitioners who had prior experience working with NPs in their practice during office hours were, in general, more willing and aware of the need to focus on more complex patients. If GPs did not change their routines of selecting patients from the presentation list in team-B, patients had to wait longer periods of time to receive treatment from a GP if their complaint did not fit the predefined NP scope of practice. This was a safety risk, especially for the more urgent patients. This is illustrated by examples provided by several team members:

Some shifts run perfectly, others you think “I wish there was a third GP now.” Especially when there are GPs who pick out patients with skin complaints from the presentation list. You get delay if more patients with abdominal pain show up. (Medical assistant, team-B).

One GP basically picked out all patients. In those cases I have to warn “think about the urgent patients”. There was an incident when an older woman got called in for a consultation, while a more urgent man was waiting to see the doctor. (Receptionist, team-B)

However, support staff indicated that the condition of the patient in the waiting room is more important than the urgency level determined during earlier triage. Therefore, medical assistants sometimes performed an extra triage in the waiting room to indicate those patients who are in urgent need of care.

Nurse practitioners in team-B did not report they treated different numbers or types of patients compared to working in team-A. An NP in team-B said: “I don’t experience any difference, you continue to treat the same complaints and that costs you the same amount of time.” In addition, NPs in team-B said they did not change their professional routines compared to working in team-A.

In both teams, team members indicated that, in general, NPs spend a longer time on consultations than GPs. Nurse practitioners and GPs assumed this is because NPs take a more extensive patient history and document more in the patients’ medical records. A GP from team-B said: “I must say, a patient with, for example, a sore, I finish those consultations within a minute and I feel confident to do that. I think NPs are more careful and still take a full history just to be sure they do their work properly.” An NP in the focus group explained; “I think we are more anxious about making mistakes, due to the vulnerable position of NPs in a relatively new profession. I think we take more extensive histories and physical exams. That might also be influenced by less experience.” Moreover, NPs reported they provide more education and counselling to patients.

Both NPs and medical assistants indicated that NPs ask less often for support from the medical assistant compared to GPs; for example, regarding putting on bandages after suturing: “GPs are used to having medical assistants in their practices that take over a lot of tasks. They ask more easily “do you want to take a look at this?” From my experience, NPs more often complete things themselves.” (Medical assistant, team-B).

Lastly, sharing the same practice ideology was not mentioned by any of the participants. They said during the shift they focus on the patients in their own surgery room and lack insight into the other professionals’ patients and treatments.

### Theme 3. Communication concerning professional roles

Although GPs and NPs worked in close proximity (consultation rooms are next to each other), both GPs and NPs said, regardless of team structure, there was little communication between them during the shift. A GP in team-A said: “I haven’t spoken to the NP all day, I started directly doing consultations with patients.” Occasionally, they had face-to-face consultation about patients, but GPs and NPs did not feel they delivered care to the patients as a team. An NP in the focus group said: “No, I can’t say it really feels like a team. We work too individualistic. I think you should speak to each other when things are going wrong.” Nurse Practitioners said they communicate with the GP regarding which patient types are excluded from NP care at the start of the shift. Most GPs, especially those whose shift started later in the day, said there were no specific work arrangements communicated about who treats which patients.

If you all start at 8h, you introduce yourself to the other team members. That didn’t happen today because all doors were already closed and you’re not gonna knock. In that case I just start my work, it’s more practical. (General practitioner, team-A).

At the start of the shift I say “I don’t treat those patients.” That’s it, then you start doing consultations. Making agreements with the GP who starts later isn’t really working, you’re either busy yourself, or the door of the GP is already closed. (Nurse practitioner, focus group).

### Theme 4. Trust and support in NP practice

The role of the NP was well accepted by receptionists and medical assistants in both teams and they had a positive view about the quality of care delivered by NPs. They also had the impression that patients are satisfied with NP care. As a medical assistant in team-B said: “From my personal experience, I don’t care if I have to work with 2 NPs, or one, or with only GPs.” There were, however, differences among GPs in support for the role of the NP. A receptionist in team-B said: “It’s hard to generalise; the one GP is fine with NPs, the other one sees problems immediately. It’s just another point of view.” Most GPs believed NPs are well capable of treating the patients within their scope of practice. Some even considered the NP being more skilled for certain types of patients than GPs. One GP from team-A explained: “I must say, it’s nice to work with NPs, they have a lot of knowledge in their field of practice. I even asked some advice from the NP today about a stoma, that was helpful.” However, some GPs believed care provided by NPs is of less quality compared to the care delivered by GPs, and certain GPs resisted the role of NPs. One GP in team-B said: “I totally disagree that they try to transform nurses into some sort of GP.” Often, these GPs expressed misconceptions about NPs’ education and legislation governing NP scope of practice. None of the GPs worried about becoming deskilled in treating certain complaints. Even though GPs treated more complex patients when there were more NPs part of the team, in both teams, GPs said that most complaints they treated were still of low complexity.

## Discussion

### Statement of principal findings

In this study we explored collaboration in teams of NPs and GPs in out-of-hours care. Several important themes emerged in the study and include lack of clarity regarding the NP role and regulations; differences in support for the NP role; variety in team members in sharing caseloads and use of skills; and limited communication during the day. These themes have important implications for collaboration in teams as well as for patient care and outcomes. For example, if team members, both including NPs and GPs, did not change their professional routines in terms of selecting patients according to their scope of practice, then it may lead to delays in patient care. We also found that in this unique care setting, collaboration between NPs and GPs did not develop over time as these providers do not have opportunities to work with each other. The environment in the GPC did not create a conducive environment for collaboration. Both NPs and GPs seem to deliver care individually with limited opportunities to communicate and share patient information with each other. Moreover, we found that GPs experienced a somewhat more complex workload if there were more NPs in the team; however, this was not a consistent pattern across all participants.

### Comparison with other studies

Consistent with findings from other studies, there was a lack of understanding of the NP role which was a major challenge for effective collaboration. Nurse Practitioners’ scope of practice excluded care of certain type of patients, which should receive care from GPs; however, GPs were not clear which types of patient should receive care from NPs. A lack of insight into the NPs’ role resulted in stagnation in effectively sharing of patient caseload between NPs and GPs, and was shown to increase the risk of delay in care for those patients outside the NPs’ scope of practice [13, 27, 28]. Therefore, role clarification of the NP is extremely important; however, it might not be adequate to focus only on the NP role [29]. In order to promote effective collaboration, it is important to assure that the roles and scope of practice of each members on the team are clear and well-understood, especially in out of office hours care models when team structures that constantly change.

When considering how to effectively deploy NPs, it is important to rethink the role of all team members [30]. First, to assure optimal patient flow for all patients, GPs should provide care to patients whose care is outside NPs’ scope of practice. Although the effect of NPs’ deployment on the workload of GPs remains unclear in the literature, most GPs in the current study indicated that a larger number of NPs in the team led to an increase in complexity of the GPs’ workload during the shift [31]. However, NPs taking on more shifts at the GPC reduced GPs’ workload in terms of reducing the number of shifts each GP worked.

Next, some participants reported that NPs took longer time for consultations which sometimes led to delays in patient care. There were two explanations for longer consultation times: one that NPs take longer time to educate patients and take detailed histories, and second, that NPs do not ask for support in a manner that GPs do [32–34]. It is important to explore NP consultation to better understand how to help NPs to be more effective in the use of consultation time. One approach could be to encourage NPs to ask for support so their time can be freed to care for complex needs of patients. Decreasing consultation times by taking less extensive histories or examinations might be inadvisable because premature closing and overconfidence are cited as major biases associated with diagnostic and treatment errors in studies among physicians [35]. Moreover, more extended health counselling is likely to improve patient outcomes [36].

Lastly, especially in teams with a smaller number of GPs and an increased risk of patient delay, medical assistants play a significant role in the protection of patient safety issues. Their knowledge and expertise are important to indicate urgent patients and assign them to the proper care provider in a timely manner.

Another finding emerging from the study was that the effect of collaboration on patient outcomes depended on the ratio of GPs to NPs in the team. This was caused by the predefined scope of practice of the NPs, whereby some patients could only be treated by GPs. In teams with one NP and three GPs, team members indicated there were enough GPs to care for patients without any delay to those patients who are outside NPs’ scope of practice. It was anticipated that this teams with this structure could care for all patients in a timely matter since previous research showed that only 23% of all patients attending out-of-hours are outside NPs’ scope of practice [4]. Although, based on these numbers, teams with two GPs and two NPs also provide enough capacity, there was an increasing risk of patient delay in teams with this structure if team members did not carefully select patients from the presentation list according to their scope of practice. Thus, team members should identify patients’ needs collectively, and the two GPs need to focus on those patients who can only be treated by a GP. However, team members indicated that overall providers cared for patients during the shift individually, rather than as a team.

In large scale organisations such as GPCs, creating involvement and acceptance of all team members is difficult [37]. The size of the organisation influences the time period to establish a collaborative relationship. Due to the ever changing team members in shifts at GPCs, team members are not well-acquainted with each other; consequently, collaboration hardly developed over time [18]. Therefore, when integrating NPs into teams, managers of the GPCs have to be aware of the need to continuously provide education, resources and support to all team members [17]. Another approach for GPC is to make separate presentation lists for GPs and NPs. Triage nurses at the call centre should be educated to guide patients to the right provider based on the patients’ complaints. If doing so GPCs less depend on GPs’ willingness to take care of patients outside NPs’ scope of practice. This has however important implications for the education and guidance of triage nurses.

There are also factors outside GPCs’ direct reach that are important to establish sustainable collaborative practice in out-of-hours care over time. First, the deployment of NPs in out-of-hours care should go hand-in-hand with their deployment during office hours. Familiarity during office hours will increase understanding and acceptance of the NP role and willingness to collaborate also during the shifts out-of-hours [38]. GPs who work with NPs in regular office hours may have favourable perceptions about NPs, which lead to a better collaboration in out of office hours. Although evidence is limited, inter-professional education might be a promising tool to equip future health-care providers for effective collaboration in complex health-care teams. This might help health delivery models such as the GPC to establish stabile collaborative practice even though team members differ in shifts and are not familiar with each other [39, 40].

### Limitations

The study has several limitations. The study relied on responses of participants and some participants might not feel comfortable sharing their views with the interviewer. Also, some NPs might not share their views in focus groups. Focus group data of GPs is not available, although it might have given additional information to enhance the findings of the individual interviews. Another limitation of the study includes the single-centre character of the study. Even though GPCs exist in most western countries, there are other organisational models delivering of out-of-hours care [3, 4]. The size of the organisation and the number of shifts of the professionals may particularly influence team collaboration. Also, NPs’ scope of practice is likely to affect team collaboration. Teams in organisations and countries where NPs have a different scope of practice might have different experiences and outcomes in collaboration [41]. Internationally lack of consensus exists on job description, skills and educational background for NPs in primary care during and out of office hours has implications for the generalisability of the current findings [5, 6].

## Conclusions

In this study we explored collaboration between NPs and GPs in out of office hours care. We found that several factors determined collaboration and included differences between GPs in support for the NP role; variety in sharing caseloads and the use of skills; and limited communication regarding collaboration during shifts. Lack of collaboration was more likely to result in patient delay in teams with more NPs. Due to a large number of GPs who work shifts at GPCs, the implementation of collaborative hardly developed over time. Enhancement of collaboration by communicating roles and making work agreements should therefore be continuously on the agenda.

## Abbreviations

ED: Emergency department
GP: General practitioner
GPC: General practitioner cooperative
NP: Nurse practitioner
